# Youth at risk of physical inactivity may benefit more from activity-related support than youth not at risk

**DOI:** 10.1186/1479-5868-3-5

**Published:** 2006-03-28

**Authors:** Kirsten Krahnstoever Davison, Dorothy L Schmalz

**Affiliations:** 1 Department of Health Policy, Management and Behavior, University at Albany (SUNY), Albany NY, USA; 2 Department of Parks, Recreation, & Tourism Management, Clemsen University, Clemson, SC 29634-0735, USA

## Abstract

**Background:**

This study examines whether associations between activity-related support and adolescents' physical activity differ for adolescents at high versus low risk of physical inactivity.

Methods:

Participants included 202 middle-school-aged girls (N = 92) and boys (N = 110). Physical activity was assessed using three self-report questionnaires. Activity-related support from mothers, fathers, siblings, and peers was assessed using the Activity Support Scale. Perceived sport competence was assessed using the Physical Activity Self Description Questionnaire. Participants' height and weight were measured and used to calculate their age- and sex-adjusted Body Mass Index percentile. Participants were classified as being at high risk for physical inactivity if they fulfilled two of the following three criteria: (1) overweight; (2) female; or (3) having low perceived sport competence.

Results:

Activity-related support from all sources was associated with higher levels of physical activity among adolescents. A stronger association between activity support and physical activity was found for adolescents at high risk for physical inactivity in comparison to adolescents at low risk.

Conclusions:

Findings from this study suggest that the activity-related support from family and friends may be an effective tool in promoting physical activity among youth at risk of physical inactivity.

## Background

Numerous studies point to the potential benefits of activity-related support from significant others on physical activity levels among youth. In particular, research to date has shown that children and adolescents are more likely to be physically active when their parents and friends are active [1-5], encourage them to be active [6-8] and participate in sport or physical activity with them [1,2,6,9,10]. Furthermore, children show higher levels of physical activity when their parents take them to places where they can be active, enroll them in organized activities and pay the associated fees [1,2,11,12]. This body of research suggests that activity-related support from family and friends is important to consider as a mechanism to promote physical activity among youth.

Before designing and implementing support-based intervention programs, however, more information is needed on whether activity support is equally beneficial to all youth. Few studies to date have examined whether the association between activity support and physical activity differs based on certain individual characteristics and in particular whether some individuals benefit more from support than others. Such information is important in order to determine whether interventions should be administered to all youth or focused on "high risk" youth.

Results from intervention research suggest that "high risk" youth may benefit more from physical activity interventions than "low risk" youth[13,14]. For example, in a study assessing the effectiveness of a physical activity intervention among girls, Boyd and Hrycaiko[13] found that girls with low self-esteem and low perceived self-competence most benefited from the intervention. The goal of this study is to examine whether youth at risk of physical inactivity are disproportionately responsive to activity-related support from family and friends relative to youth who are not at risk. That is, this study assessed whether risk status moderates the association between activity support and physical activity.

A number of individual characteristics have been linked with physical inactivity, or low levels of physical activity, among youth. Girls [15,16], youth who are overweight[17], and youth with low perceived sport competence [18,19] are less active than their respective counterparts. Moreover, these risk characteristics tend to co-occur such that girls [14] and overweight [20,21] youth report lower perceived sport competence than boys and non-overweight youth respectively. Hence, it appears that youth at high risk for physical inactivity experience multiple risk factors. With this in mind, the current study examines the effect of exposure to multiple risk factors on the association between activity-related support and physical activity. Adolescents are classified as being at high risk of physical inactivity if they experience two or more of the aforementioned risk characteristics (i.e., female, overweight, low perceived sport competence). It is predicted that a stronger association will be identified between activity support and physical activity among high risk youth in comparison to low risk youth, suggesting that high risk youth are disproportionately responsive to activity support.

## Methods

### Participants

Participants included 202 middle school girls (N = 92) and boys (N = 110) in grades 6 to 8, who were recruited from a middle school in a rural community in central Pennsylvania. The mean age of participants was 12.7 ± .8 years for boys and 12.5 ± .8 years for girls. Data were collected in fall of 2002. The study was reviewed and approved by the Ethics Review committee of the associated university. Signed parental consent and participant assent were required in order to participate in the study. In-class questionnaires were administered by the authors and two research assistants. Participants' responses were confidential.

### Measures

#### Physical activity

Three self-report measures were used to assess participants' physical activity including the Children's Physical Activity scale, an activity checklist and the physical activity subscale of the Physical Self Description Questionnaire. The Children's Physical Activity scale (CPA) was used to assess general tendency or inclination to be physically active[22] (e.g., "I like to exercise or be physically active more than anything else"; "I would rather watch TV or relax inside than be active outside"). The CPA contains 15 items and uses a 4-point response scale. Scores across all items were averaged to provide a measure of girls' total inclination toward, or preference for, physical activity. Previous research using a sample of 9 and 10 year old girls supports the predictive validity of the CPA [22]; specifically, scores on the CPA were found to be positively related to 1-mile run/walk time and negatively related to percentage body fat and Body Mass Index. The internal consistency coefficient for the current study was α = .80.

An activity checklist was used to assess participation in activities on a regular or competitive basis. Participants were presented with a list of 28 activities (e.g., basketball, rollerblading, tennis) and were asked to indicate whether they participated in the activity competitively or on a regular basis (30 min 3 times/week) within the past year. The total number of activities selected was summed to reflect the total number of sports and competitive activities participants were involved in over the past year. Although there are no validity data available for the activity checklist, activities included on the checklist are consistent with preferred activities as reported by rural youth. Specifically, the most preferred activities among rural youth, as identified by Savage and Scott [23], include tennis, volleyball, swimming, softball, bicycling, football, ice skating, backpacking and hiking, and weightlifting. With the exception of back-packing and hiking, all of these activities were included on the activity checklist.

Finally, the physical activity subscale of the Physical Self Description Questionnaire (PSDQ)[24] was used to assess general levels of physical activity. This subscale of the PSDQ includes 6 items and uses a 6-point response scale (e.g., "several times a week I exercise or play hard enough to breathe hard or sweat"). Scores across the 6 items are averaged to create a generalized measure of physical activity. Previous research, based on a sample of high school students (12–18 years), supports the psychometric properties of the PSDQ, including its test-retest reliability and convergent and discriminant validity [24]. Additional research, also using a sample of high school students (aged 13–15 years), found that scores on the physical activity subscale are significantly correlated with the number of hours of physical activity during a typical week and one mile run time[18]. The internal consistency co-efficient for this study was α = .87.

Physical activity is a complex multidimensional construct [25]. Therefore, a summary physical activity score was created based on all three self-report measures of physical activity using principal component analysis. The total score for each of the three measures of physical activity was entered into the analysis and a single principal component was outputted based on these variables. Conceptually speaking, in principal component analysis, all variables are converted to a standard metric with a mean of 0 and a standard deviation of 1 and combined to form a single score using weights (or factor loadings) that reflect the intercorrelations between the measures. This procedure ensures that highly correlated measures contribute to the principal component to a greater extent than less correlated measures. Creating an activity score based on multiple measures is more representative of general levels of physical activity and reduces the error involved in its measurement [26,27]. In addition, reducing the number of measures used decreases the number of analyses performed and the likelihood of a type I error. All measures were highly and significantly correlated with the summary score, with no one measure dominating the summary score (CPA r = .83; PSDQ r = .80; sport participation r = .63; p <.01). Hence, the summary measure of physical activity (mean = 0, std = 1, range = -3.7 – 2.9) represents an inclination to be active (CPA), generalized levels of physical activity (e.g., PSDQ), and participation in organized activities (e.g., sport participation). This variable was normally distributed.

#### Physical activity support

The Activity Support Scale[2] is a 27-item scale that assesses activity-related support from mothers, fathers, siblings and peers. Forms of support that are assessed by the Activity Support Scale include, for example, adolescents' reports that their parents drive them to and from sporting events and include them in their sporting or exercise routines, that their friends do active things with them and admire people who are physically active, and that their siblings are active and motivate them to be active as well. Although the measures of maternal and paternal support include subscales assessing logistic support (e.g., making arrangements so their child can be physically active) and modeling (doing activities with children), total maternal and total paternal support (which combines scores for logistic support and modeling) are used in this study to reduce the number of analyses performed. For maternal and paternal support, participants completed the measure with reference to their parent, stepparent or legal guardian. For the sake of simplicity, however, this group is collectively referred to as "parents". The factorial structure, internal consistency (α = .71 – .76) and predictive validity of the Activity Support Scale in this sample have been previously reported [2].

#### Weight status

Participants' height (to the nearest quarter inch) and weight (to the nearest .1 of a pound) were measured by the school nurse. Participants were measured without shoes in light clothing (i.e., shorts and t-shirt). Values for height and weight were used to calculate participants' Body Mass Index (weight(kg)/height(m)^2^). Age- and gender-specific BMI percentile scores were then calculated using the 2000 CDC growth charts [28]. In this study, adolescents who were at risk of overweight or who were overweight were examined as a single group (i.e., BMI percentile ≥ 85) [28]. To simplify the presentation and discussion of results, this group is referred to collectively as overweight.

#### Perceived sport competence

In addition to measuring general levels of physical activity, the PSDQ was used to measure perceived sport competence [24]. The sport competence subscale of the PSDQ includes 6 items and uses a 6-point response scale. In addition to the general measurement qualities of the PSDQ outlined above, scores on the sport competence subscale are associated with higher levels of physical activity, higher strength and greater endurance [18]. The internal consistency co-efficient for this study was α = .93.

#### Classification of risk groups

Information about participants' gender, weight status, and perceived sport competence was used to classify whether they were at high versus low risk of physical inactivity. Classifications for each of the identified risk variables were collapsed to give a measure of exposure to multiple risk factors. Specifically, participants were classified as being at high risk for physical inactivity if they fulfilled two out of three of the following criteria: (1) female; (2) overweight; or (3) having low perceived sport competence (based on a mean split). The inclusion of gender in the risk classification was warranted because, although boys consistently report higher levels of physical activity than girls, previous research with the same sample has shown that there are no gender differences in exposure to activity-support [2]. As a result, any differences identified in the association between support and physical activity for the high versus low risk group will not unduly reflect gender differences in exposure to activity support.

#### Analyses

Analyses were performed using SAS version 8.02 (Cary, NC). Background characteristics including family income, parent education and family composition were assessed as potential confounding variables. In instances in which a background characteristic was associated with the independent and dependent variable, it was entered into the analysis of interest as a covariate. Differences in physical activity and activity-related support were assessed for low and high risk youth using independent t-tests (see Table 1). The moderating effect of risk classification on the association between activity-related support and adolescents' physical activity was assessed using multiple regression analysis. A separate model was run for each source of support. For each analysis, the main effect of risk group (high versus low), the main effect of activity support, and the interaction between risk group and support were entered into the model predicting adolescents' physical activity (see Table 2). The cumulative effect of activity support from mothers, fathers, siblings and peers on adolescents' physical activity was also examined for each risk group using multiple regression analysis.

**Table 1:** Mean (SD) physical activity and activity-related support for adolescents at high versus low risk of physical inactivity.

	Low Risk (N = 122)	High risk (N = 80)	t-value	Effect size^† ^(Cohen's d)
Physical activity	.39 (1.08)	-.61 (1.46)	5.45**	.78
				
Source of social support				
Mother	2.74 (.63)	2.72 (.57)	0.14	.03
Father	2.90 (.62)	2.71 (.68)	1.93*	.29
Sibling	2.77 (.74)	2.62 (.68)	1.41	.21
Friend	3.32 (.58)	3.08 (.54)	3.03**	.43

Note: Scores for physical activity are standardized with a mean of 0.

* p < .05 ** p < .01

^† ^According to Cohen [37] an effect size of .2 is considered a small effect, .5 is a medium effect, and .8 is a large effect.

## Results

### Sample characteristics and preliminary analyses

Participants were predominantly non-Hispanic white (97%) and were from families with low to middle levels of income and education. Specifically, the percentage of families with a combined family income of (a) <$20,000, (b) ≥$20,000 and < $50,000, or (c) ≥ $50,000 was 29%, 57%, and 14% respectively. In addition, some high school or a high school diploma was reported as the highest level of education for 56% of the fathers and 45% of the mothers. Forty five percent of girls and 41% of boys were classified as being overweight, which is consistent with reported rates of overweight among rural children and adolescents [29-31].

For maternal support, 95% of participants responded with reference to their mother, 1% with reference to their stepmother and 4% with reference to their grandmother or female. For paternal support, 83% of participants responded with reference to their father, 17% with reference to their stepfather, 6% with reference to their grandfather or male guardian, and 5% reported having no male figure in their life. Preliminary analyses examined family composition (which was inferred from the aforementioned information) as a potential confounding variable. Participants who responded with reference to their mother and their father (i.e., participants from two parent families) reported significantly higher paternal support than participants who responded with reference to a stepparent, grandparent or guardian. Family constellation, however, was not linked with risk status or physical activity. Therefore, it was not necessary to include family composition as a covariate in the analyses. Similarly, family income and education were not associated with the independent and dependent variables (i.e., were not identified as confounding variables) and therefore were not entered into analyses as covariates.

### Profile of the high risk group

Of the 202 participants in the study, 80 were classified in the high risk group (40%) and 122 in the low risk group. Among participants in the high risk group, 76% were female, 76% were overweight, and 72% reported below average gender-specific perceived sport competence. The corresponding figures for the low risk group were 27%, 20% and 21%. No combination of risk characteristics was disproportionately represented in the high risk group. Of the 80 adolescents classified as high risk, 19 (23.7%) had all three risk characteristics (female, overweight, low perceived competence), 23 (28.7%) were female and overweight, 19 (23.7%) were female and reported low sport competence, and 19 (23.7%) were overweight and reported low sport competence (and were male).

### Links between adolescents' risk status, activity-related support and physical activity

Differences in physical activity and activity support were assessed for adolescents at high versus low risk of physical inactivity (see Table 1). Adolescents in the high risk group reported significantly lower levels of physical activity than adolescents in the low risk group. In addition, high risk adolescents reported significantly lower levels of activity support from fathers and friends than low risk adolescents. No differences for high versus low risk adolescents were noted in activity support from mothers or siblings.

Table 2 presents the results from the regression analyses assessing the moderating effect of risk status on the association between activity support and physical activity. For each source of support, there were significant main effects of risk and support. As predicted, however, the association between activity support and physical activity was moderated by risk status. The interaction term was marginally significant for paternal support (p = .10) and significant for maternal, sibling and peer support (p < .05).

**Table 2:** Results from the regression models predicting adolescents' physical activity

Parameters from each regression model	β (SE)	R^2 ^for model
Main effect of risk	-.37 (.17)**	.23**
Main effect of **maternal **support	.12 (.10) **	
Interaction between risk and **maternal **support	.23 (.18) **	
		
Main effect of risk	-.31 (.17) **	.26**
Main effect of **paternal **support	.25 (.12) **	
Interaction between risk and **paternal **support	.14 (.18) †	
		
Main effect of risk	-.33 (.17) **	.24**
Main effect of **sibling **support	.16 (.12) **	
Interaction between risk and **sibling **support	.21 (.18) **	
		
Main effect of risk	-.25 (.17) **	.33**
Main effect of **peer **support	.34 (.11) **	
Interaction between risk and **peer **support	.22 (.17) *	

Note: † p < .10 * p < .05 ** p < .01

High risk coded as 1, low risk coded as 0.

The beta-weight for risk is slightly different across models because a different source of support was included in each model. The resulting beta weight is the independent effect of risk.

The figure illustrates the nature of the significant interaction effects. Scores for physical activity were plotted for different combinations of risk status and support. Specifically, the mean physical activity score was calculated for 4 groups: low risk and low support (lower quartile for support); low risk and high support (upper quartile for support); high risk and low support; and high risk and high support. As shown in the figure, the extent to which the mean scores differ for the low and high risk groups depends on their exposure to activity support. Specifically, the difference in the physical activity scores for the low and high risk groups is quite pronounced under conditions of low support, with the high risk group reporting particularly low levels of physical activity. In the presence of high support, however, this difference is greatly reduced with the high risk group reporting a level of physical activity almost comparable with the low risk group.

The cumulative effect of activity support from all sources on adolescents' physical activity was also assessed for high and low risk youth. Results from the regression analyses showed that support from mothers, fathers, siblings and peers collectively explained 40% of the variance in physical activity among adolescents in the high risk group (F (4,74) = 11.81, p < .0001) and 21% of the variance in physical activity for adolescents in the low risk group (F (4,115) = 7.39, p < .0001).

## Discussion

Previous research has shown that activity-related support is positively associated with physical activity levels among youth. Results from this study build on previous research by showing that the link between activity-support and adolescents' physical activity differs for youth at high versus low risk of physical inactivity. As hypothesized, associations between activity support and physical activity were stronger for adolescents at high risk, which included girls, overweight youth and youth with low perceived sport competence, than low risk youth. While differences in reported physical activity were substantial for low and high risk youth under conditions of low support (with low risk youth reporting higher levels of physical activity) physical activity levels were comparable among the risk groups under conditions of high support. This pattern of findings was evident for all sources of support with the exception of paternal support, for which the interaction between support and risk was a trend. The less pronounced "benefits" of paternal support among high risk youth may be explained by the fact that high risk youth reported significantly less support from their fathers than low risk youth. Overall, results from this study suggest that youth at risk of physical inactivity may be disproportionately responsive to activity-related support than youth at low risk.

There are a number of reasons why youth at risk of physical inactivity may be more responsive to activity support compared to youth at low risk. First, youth at low risk may be more intrinsically motivated to be active, which may be driven by greater athletic ability and greater success in, and enjoyment of, physical activity. External forms of support are less likely to be helpful in this situation because low risk youth may be active regardless of peer and familial support. In contrast, youth at high risk may depend more on external support due to a lack of intrinsic motivators and potentially fewer opportunities to be active. In addition to differences in sources of motivation to be active, youth at high risk of physical inactivity may be exposed to lower levels of support than youth at low risk. Indeed in this study, youth at high risk reported significantly lower support from fathers and friends than youth at low risk. While no definitive statements can be made about the defining characteristic of the high risk group that was associated with lower paternal and peer support, previous research suggests that this was not simply a reflection of the large percentage of girls in the high risk group. Specifically, previous research[2] using the same sample showed no gender differences in exposure to activity support from parents or peers. Thus, it is likely to be a combination of factors that is linked with lower exposure to support.

Findings from this study suggest that it may be beneficial for family-based activity promotion programs to target high risk youth. Such interventions should focus on helping parents to identify whether their children are at risk of inactivity and in such situations make a special effort to become involved in their children's physical activity, create opportunities for their children to be active, and support their children's activity. Research by Davison, Downs and Birch [32] has shown that youth at high risk (i.e., with low perceived athletic competence) are less likely to elicit support from their parents than youth with high perceived competence. Therefore, it is important that parents do not wait for their children to elicit such support; they should take a proactive approach. Fathers in particular may need to make special efforts to be proactive in encouraging their children to be active. In this study, high risk group were less likely to report support from their fathers and the positive links between support and physical activity were less pronounced for paternal support in comparison to support from mothers, siblings and peers. Parents can also facilitate the provision of support from siblings by, for example, enabling a younger sibling to participate in a sporting event with an older sibling. Finally, parents need to be aware of the importance of peers in the lives of adolescents and the beneficial role that they can play in fostering their children's interest in and perseverance with physical activity and use this knowledge to their advantage to encourage their children to be active. For example, parents could help foster support within the peer network by identifying activities that their children's friends enjoy, organizing activities that can include a friend, or facilitating the ability for their children to spend time with their friends while doing something active (e.g., taking their child and his/her friends ice skating).

Key strengths of this study include its extension of previous research and its implications for the design of physical activity promotion programs. This is the first study to assess whether risk for inactivity moderates the association between activity support and physical activity among youth. Results highlight the possible benefits of targeting high risk youth in interventions aimed at increasing physical activity among adolescents. Limitations of this study include its cross sectional design, the lack of ethnic and racial diversity in the sample, the use of self-report measures of physical activity with limited measurement information, and the assessment of activity-support from a single perspective (i.e., that of the adolescent). The direction of the association between activity support and physical activity cannot be determined in this study due to its cross sectional design. While it is likely that support leads to higher levels of physical activity, it is also possible that higher levels of physical activity result in higher support. Additional longitudinal research is required to clarify the direction of this association. Future research can also build on the findings of this study by examining whether minority youth (who may be particularly at risk of physical inactivity) are similarly responsive to activity-related support from significant others, and by replicating findings from this study using an objective measure of physical activity and measures of activity support from multiple reporters including parents. Future research could also extend findings from the current study by examining whether youth at high risk of physical inactivity disproportionately reap the psychological benefits of physical activity such as improved self esteem[33].

### Summary and conclusion

Physical activity is lauded for the positive physiological and psychological benefits that it affords including decreases in body fat [34,35], improved glucose control[36], higher self esteem [33] and lower stress [33]. Therefore, identifying avenues to increase physical activity, particularly among youth at risk for physical inactivity, is an important and worthwhile objective. In this study, stronger associations were identified between activity support from parents, siblings and peers and physical activity among youth at risk of physical inactivity – including girls, overweight youth and youth with low perceived sport competence – in comparison to youth who were not at risk. Findings from this study support the development of family-based physical activity interventions that target parents of high risk youth.

## Competing interests

The author(s) declare that they have no competing interests.

## Authors' contributions

KKD designed the study, ran the analyses and drafted the manuscript. DS oversaw data collection and provided editorial comments on the manuscript.

**Figure 1:**
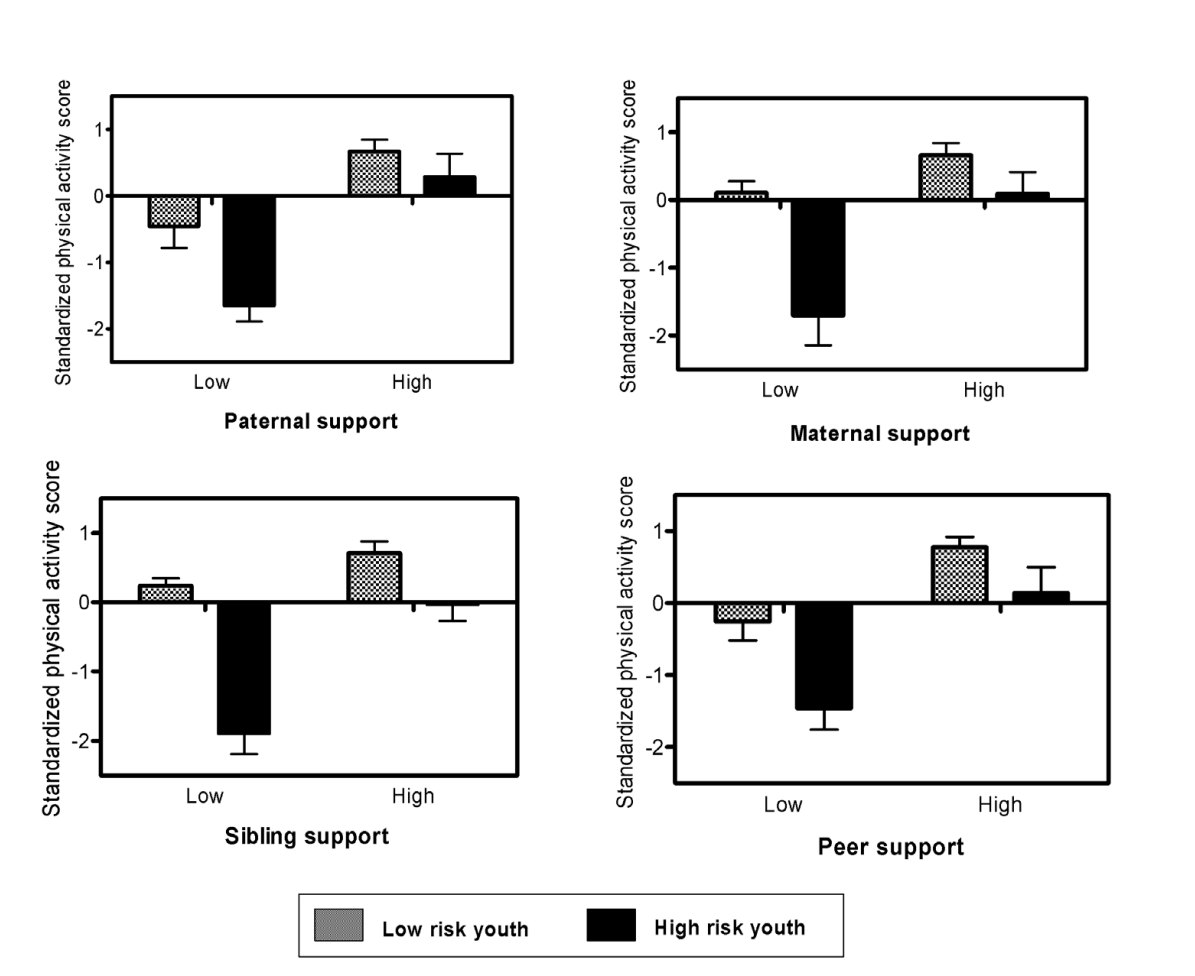
Mean (SE) standardized physical activity score for youth in the high and low risk groups exposed to high and low levels of support. Note: High support = upper quartile for support, Low support = lower quartile for support; High risk youth ≥ 2 risk factors for low physical activity, Low risk youth < 2 risk factors for low physical activity (risk factors include being female, being overweight, and having low perceived sport competence)
